# Supplementary material for: When do apples stop growing, and why does it matter?
Source: PLoS One. 2021 Jun 10;16(6):e0252288. doi: 10.1371/journal.pone.0252288 (PMC8192000; doi:10.1371/journal.pone.0252288)
Supplement: Attachment — Submitted filename: Response to Reviewers.docx [file pone.0252288.s001.docx]

Response to Reviewers:

Reviewer #1: I have reviewed the submission "When do apples stop growing, and why does it matter?" authored by Maria D Christodoulou and colleague. In this manuscript, the authors explored the developmental process of apples during the growing season with respect to size morphometric, specifically weight, length, diameter, and centroid size and a new ‘Log-log linear’ model is proposed according to these data. Importantly, a positive correlation between the variables, where a 10% increase in growing degree days is associated with a 1% increase for the predicted morphometric, can be found by this model. It will be available to commercial distribution of apples.

The manuscript is concise and well written, but I have a few questions about it.

1. As we known, biennial bearing often occurs in fruit trees and also affects fruit size. How about the fruit yield of the experimental trees at different years?

We thank reviewer #1 for their questions. Biennial patterns were a primary motivation for running the trial for two consecutive years for half the cultivars in our study. As part of our preliminary analysis, we did compare the weight of the fruit in the harvest weeks for each cultivar between the two years using a Welch’s 2 sample t-test and found no significant differences between the two years. We have now updated our code script to include this step of our preliminary analysis. We have also amended lines 185-188 of our manuscript to state this. What we did not record during the sampling was whether there were overall substantial differences in fruit numbers on the trees between the years and therefore we cannot comment on the overall yield.

2. I suggest that authors should add some detailed information about the meaning of different growth curve (Linear, Sigmoid, Curvilinear or else mentioned in this research) in the introduction.

We have included a paragraph with a more detailed description of the overall shapes presented in the literature in lines 113-124 of our Introduction.

3. If author can give a discussion about the limitation of this model.

We have included a segment on the weak points of the log-log linear regression in lines 223-228 as per the reviewer’s suggestions.

Reviewer #2: The manuscript which is named “When do apples stop growing, and why does it matter?” analyzed twelve apple cultivars at regular time points throughout the growing season with four morphometrics, and finally get a conclusion that a 10% increase in growing degree days is associated with a 1% increase for the predicted morphometric. This research is very interesting which could provide suggestions when to pick apples to balance the storage and maturity. Thus, I recommend this work should be accepted for publication, but the results parts wrote too simplified and should be improved.

We thank reviewer #2 for their feedback and recommendation for publication. We are not clear as to what their precise feedback on the improvement of our results is as they follow closely the steps of our analysis.
